# Small RNA-directed epigenetic programming of embryonic stem cell cardiac differentiation

**DOI:** 10.1038/srep41799

**Published:** 2017-02-06

**Authors:** Hossein Ghanbarian, Nicole Wagner, Jean-François Michiels, François Cuzin, Kay-Dietrich Wagner, Minoo Rassoulzadegan

**Affiliations:** 1 Biotechnology Department, School of Advanced Technologies in Medicine, Shahid Beheshti University of Medical Sciences, Tehran, Iran; 2 Université Côte d’Azur, CNRS, INSERM, IRCAN, 06107, Nice, France; 3 Université Côte d’Azur, CNRS, INSERM, iBV, 06107 Nice, France; 4 Department of Pathology, CHU Nice, Nice, France

## Abstract

Microinjection of small noncoding RNAs in one-cell embryos was reported in several instances to result in transcriptional activation of target genes. To determine the molecular mechanisms involved and to explore whether such epigenetic regulations could play a role in early development, we used a cell culture system as close as possible to the embryonic state. We report efficient cardiac differentiation of embryonic stem (ES) cells induced by small non-coding RNAs with sequences of *Cdk9*, a key player in cardiomyocyte differentiation. Transfer of oligoribonucleotides representing parts of the *Cdk9* mRNA into ES and mouse embryo fibroblast cultures resulted in upregulation of transcription. Dependency on Argonaute proteins and endogenous antisense transcripts indicated that the inducer oligoribonucleotides were processed by the RNAi machinery. Upregulation of *Cdk9* expression resulted in increased efficiency of cardiac differentiation suggesting a potential tool for stem cell-based regenerative medicine.

We reported earlier that microinjection of small non-coding RNAs is associated with epigenetic modifications and results in transcriptional activation of specific target genes^1^,^2^,^3^. Whether epigenetic mechanisms are involved in the initial determination of gene expression in the early embryo is an important question. Differentiation of cardiomyocytes is an early event during embryogenesis *in vivo*, which can be monitored by the appearance of beating cells in cultures *in vitro*^4^. To promote cardiac differentiation of ES cells, we attempted to modulate expression of *Cdk9*, one of the main actors of cardiac differentiation *in vivo*. We reported previously that transfer of small noncoding RNAs homologous to the *Cdk9* transcript or of a cognate microRNA into one-cell embryos led to transcriptional activation of *Cdk9* and hypertrophic growth of cardiomyocytes^2^. As a first step toward understanding how the epigenetic change is initiated in early embryonic cells upon transfer of the sequence-homologous oligonucleotides, we tested whether the effect could be mimicked in cell culture. For comparison to the developmental changes induced by injection in early embryos, we chose murine ES cells with a full potential of derivation into differentiated cell types. As for the basic mechanisms, we confirmed that changes in transcriptional activity were induced by homologous small RNAs in ES cells as in the early embryo and as in established cell lines^5^,^6^,^7^. We evidenced a role of antisense transcripts of the locus and a requirement for Argonaute proteins. We further observed a significant biological consequence with an increased potential of the reprogrammed ES cells to differentiate into cardiomyocytes.

## Results

### *Cdk9* induction following transfer of transcript fragment (TF) in different cell systems

**Figure 1:**
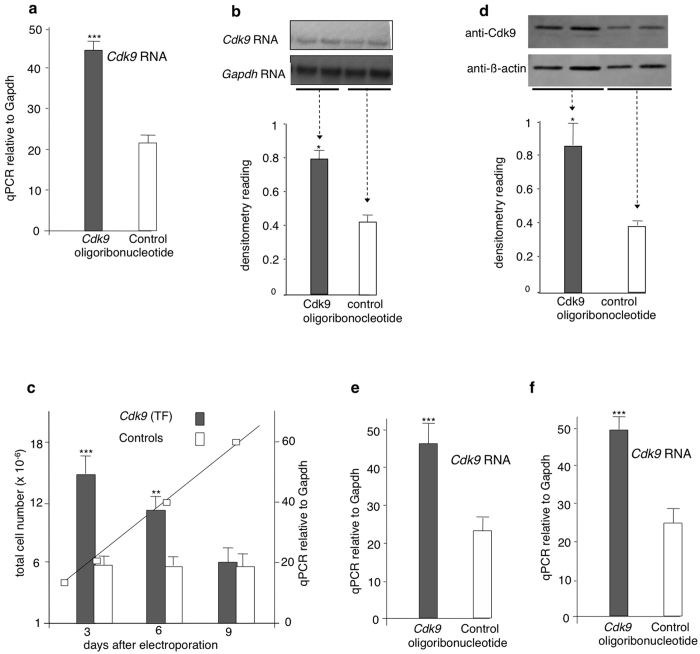
Increased expression of *Cdk9* RNA in cell systems after transfer of short transcript fragments (TF). (**a**) Increased *Cdk9* expression in ES cells was analyzed by quantitative Real-Time PCR. RNA homologous to *Cdk9* exon 7 (Cdk9-f, Table 1) was transferred by electroporation in mass cultures of the cells and quantitative RT-PCR determination of *Cdk9* RNA was performed after 2 days (*n = 5*). (**b**) Northern blot analysis shows increased *Cdk9* RNA level following TF electroporation in mass culture of ES cells. (**c**) Increased *Cdk9* expression is maintained during growth of TF-electroporated cells in culture. Average values of total cell number (straight line) and of *Cdk9* RNA measurements (bars) at successive times during cell growth and duplications. Changes in RNA levels were maintained until they returned to control levels after an estimated number of 15 duplications (n = 3). (**d**) *Cdk9* protein level in ES cells was examined by Western blot analysis 2 days after small sense RNA fragment electroporation. Increased *Cdk9* expression was also detected in Mouse Embryonic Fibroblasts (**e**) and a human keratinocyte cell line (**f**) following TF electroporation (*n = 8* each). Data are mean ± S.E.M. *p < 0.05, ***p < 0.001.

RNA-mediated transcriptional upregulation of *Cdk9* and other loci had been reported previously upon microinjection of small size RNAs in fertilized eggs^1^,^2^,^3^. To investigate whether *Cdk9* target mRNAs induce transcriptional variation in other cell types, mouse embryonic stem cells were analysed after electroporation of a 22-nt oligoribonucleotide with a nucleotide sequence identical to that of the *Cdk9* mRNA in exon 7 (*Cdk9*-f, Table 1). Extracts prepared 48 hours after electroporation showed the same average increase in *Cdk9* expression (approximately 2-fold) as observed previously in embryos after microinjection (Fig. 1a,b). During further growth in culture, the elevated RNA levels were maintained until they returned to control levels after an estimated number of 15 cell divisions (Fig. 1c). A corresponding increase in protein levels was measured by Western blot analysis (Fig. 1d). Moreover, a comparable increase in *Cdk9* expression was evidenced upon electroporation of the transcript fragment into mouse embryonic fibroblasts (MEFs) (Fig. 1e) and a human keratinocyte cell line (Fig. 1f).

### Increased transcription of the *Cdk9* locus

**Figure 2:**
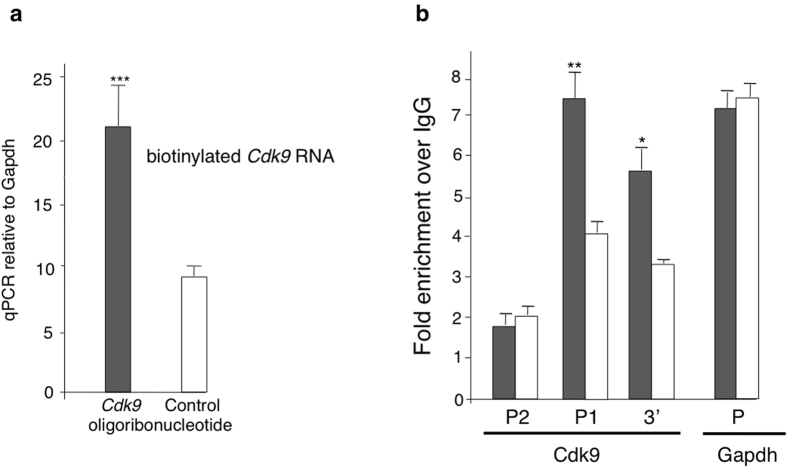
Increases in RNA levels reflect changes in transcriptional activity. (**a**) Increased levels of *Cdk9* RNA reflect the actual rate of transcription. Incorporation of biotin-UTP in permeabilized sense transcription fragment electroporated and control ES cells. Amounts of biotin-labeled *Cdk9* RNA were determined by quantitative RT-PCR on the streptavidin bound fraction (see Experimental Procedures). Data are mean ± S.E.M. of 3 independent experiments. ***p < 0.001. (**b**) Increased load of RNA polymerase II determined by ChIP analysis on the P1 promoter and the 3′ UTR, but not on the P2 promoter of the *Cdk9* locus in sense transcription fragment (Cdk9-f) electroporated and control ES cells (*n* = 4 each). *p < 0.05, **p < 0.01.

Run-on measurements of elongation rates pointed to an enhanced transcriptional activity as the cause for the increase in *Cdk9* expression (Fig. 2a). This modification might result from changes in the chromatin structure involving the locus itself. Chromatin immunoprecipitation using an antibody directed against RNA polymerase II followed by quantitative PCR revealed higher enrichment in *Cdk9*-f electroporated ES cells compared to controls in the P1 promoter region and the 3′ UTR, but not in the P2 promoter (Fig. 2b). The RNA-mediated transcriptional activation process indicated by our results reminds of RNA activation and suppression effects reported in human cells for p21, E-cadherin, and progesterone receptor genes^5^,^6^,^7^,^8^, a finding subsequently extended to other mammalian species, including the mouse^9^.

### Antisense transcription at the *Cdk9* and *Sox9* loci

**Figure 3:**
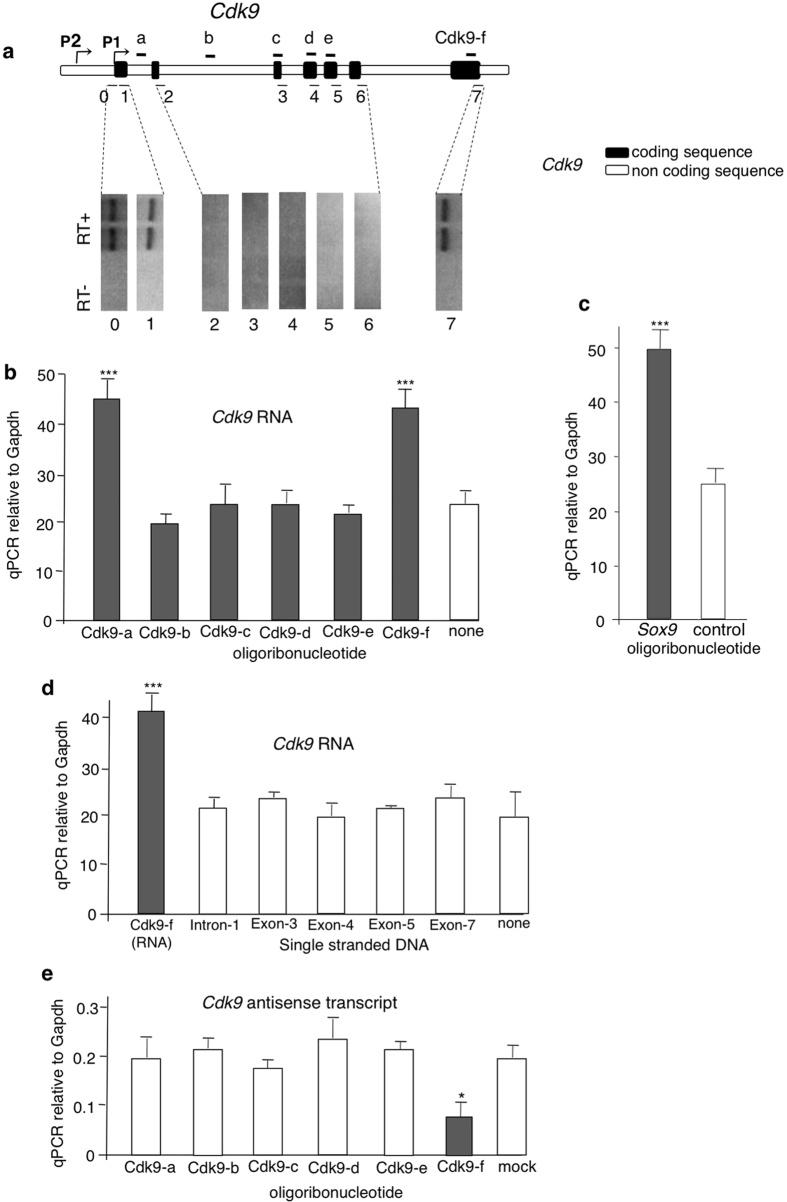
Antisense transcription at the *Cdk9* locus. (**a**) Regions analyzed for the presence of antisense transcripts throughout the *Cdk9* gene. Top: schematic representation of the locus with exons shown as closed bars. The positions of the regions analyzed are indicated. Bottom: detection of antisense RNA transcripts by strand-specific RT-PCR reactions using primers listed in Table S2. All assays were performed in duplicate, ‘‘RT-’’: reverse transcriptase omitted. The same analyses applied to the human *Cdk9* and the murine Sox9 locus are shown in Figs S2 and S3, respectively. (**b**) Levels of *Cdk9* expression following electroporation of oligoribonucleotides with exonic and intronic sequences in a region transcribed on both strands (and f regions) and sequence of regions with only sense transcription (b, c, d, and e regions). (**c**) Levels of Sox9 expression following electroporation of an oligoribonucleotide with exonic sequence. (**d**) Levels of *Cdk9* expression following electroporation of the Cdk9-f oligoribonuncleotide and several single stranded DNA oligonucleotides. (**e**) *Cdk9* 3′ antisense RNA levels in ES cells were analyzed by quantitative Real-Time PCR. The indicated RNA oligoribonucleotides (Table 1) were transferred by electroporation in mass cultures of the cells and quantitative RT-PCR determination of *Cdk9* antisense RNA was performed after 2 days (*n = 4*). Data are mean ± S.E.M. *p < 0.1, ***p < 0.001.

Specificity of transcriptional induction by transcript fragments implies a sequence recognition mechanism. The most obvious mechanism for specific locus recognition is hybridization with sequences complementary to the inducing RNAs. Antisense transcripts, a frequent feature of the mammalian genome^10^, would provide a possible explanation. Strand-specific RT-PCR assays (Fig. 3a) confirmed the presence of transcripts complementary to the most 3′ region of the mRNA and identified antisense RNAs transcribed from a 5′ region corresponding to the promoter, the first exon, and the first intron. A schematic representation of the *Cdk9* genomic locus including the antisense transcripts and the miR-1 binding site is provided in Supplemental Fig. 1. Similar analyses showed the presence of human *Cdk9* antisense transcripts in the keratinocyte cell line (Suppl. Fig. 2) and antisense transcripts covering the complete *Sox9* locus in mouse ES cells (Suppl. Fig. 3).

To investigate a potential regulatory role of antisense transcripts in the *Cdk9* locus, short single-stranded RNA oligonucleotides (antagoNATs) derived from the *Cdk9* sense strand were examined to inhibit the activity of antisense transcripts. As shown in Fig. 3b, oligonucleotides with either an intronic sequence of *Cdk9 (Cdk9*-a, Table 1) or the exonic sequence in the 3′ region (*Cdk9*-f, see Fig. 3a) induced the transcriptional activation of *Cdk9*. Both correspond to regions in which antisense transcripts are detected. This was also the case for the *Sox9*-a oligonucleotide (Table 1), whose electroporation induced upregulation of *Sox9* (Fig. 3c). Single stranded DNA oligonucleotides from the different regions of *Cdk9* (Table 1) did not induce expression of the gene (Fig. 3d). Since antisense transcripts were not detected in the central region of *Cdk9*, we were able to test the possibility that antisense RNA could be dispensable for the establishment of the epigenetic change. Sequences of the oligonucleotide *Cdk9*-b in intron 2, *Cdk9*-c in exon 3, *Cdk9*-d in exon 4, and *Cdk9*-e in exon 5 (Fig. 3a) are from regions in which no antisense transcripts were detectable. Electroporation of these oligoribonucleotides did not modify expression of *Cdk9* (Fig. 3b). Although other variables may have to be considered, this result clearly supports the notion that antisense RNAs are the targets of the sense oligoribonucleotides. Electroporation of the *Cdk9*-f oligoribonucleotide reduced expression of the complementary 3′ region antisense transcript while electroporation of the other *Cdk9* oligoribonucleotides had no effect on the expression of this antisense transcript (Fig. 3e). A siRNA directed against the antisense transcript reduced expression of the complementary 3′ region antisense transcript and induced expression of *Cdk9* (Suppl. Fig. 4).

### Argonaute proteins are required for reversible transcriptional activation by noncoding RNAs

**Figure 4:**
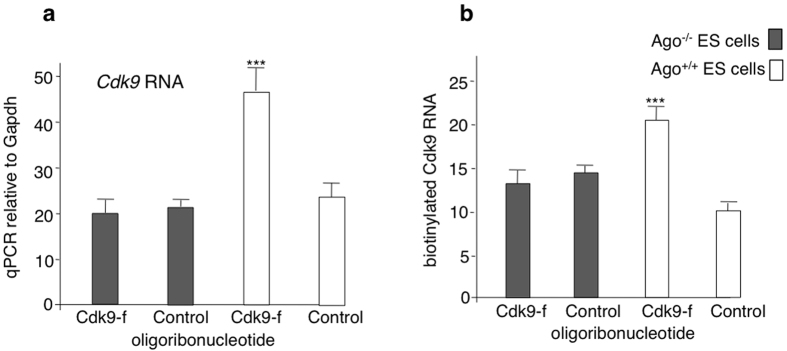
Transcriptional modification of *Cdk9* expression by a transcript fragment requires Argonaute proteins. (**a**) Cdk9-f oligonucleotides (Table 1) were electroporated in mass cultures of Ago1,3,4-deficient and of wild type ES cells. Quantitative RT-PCR determination of *Cdk9* RNA was performed after 2 days (*n = 3*). (**b**) Transcriptional activation does not occur in Ago-deficient cells. Run-on assays performed by measuring incorporation of biotin-UTP in permeabilized Ago1,3,4-negative cells and in wild type ES cells. Amounts of biotin-labeled *Cdk9* RNA were determined by quantitative RT-PCR on the streptavidin-bound fraction (*n = 3*). Data are mean ± S.E.M. ***p < 0.001.

If locus-specificity were assured by interaction of the inducer oligoribonucleotides with antisense RNAs of the locus, one would expect a role of Argonaute complexes, which can be tested in cell culture systems by using Ago-negative mutants. As shown in Fig. 4a, when electroporated into ES cells deficient for Ago1, 3, and 4 and hypomorphic for Ago2^11^, the transcript fragment did not induce an increase in *Cdk9* expression and similarly, *Sox9* transcriptional induction was not observed following electroporation of the *Sox9* oligoribonucleotide in Ago-deficient ES cells (data not shown).

The observation that transcriptional activation was not detected by run-on assays in Ago-deficient cells (Fig. 4b) confirmed a causal relationship between transcriptional activation and the Ago-dependent state that we assume involves the sense oligoribonucleotides and reverse transcripts.

### Locus specificity of RNA-mediated gene activation

**Figure 5:**
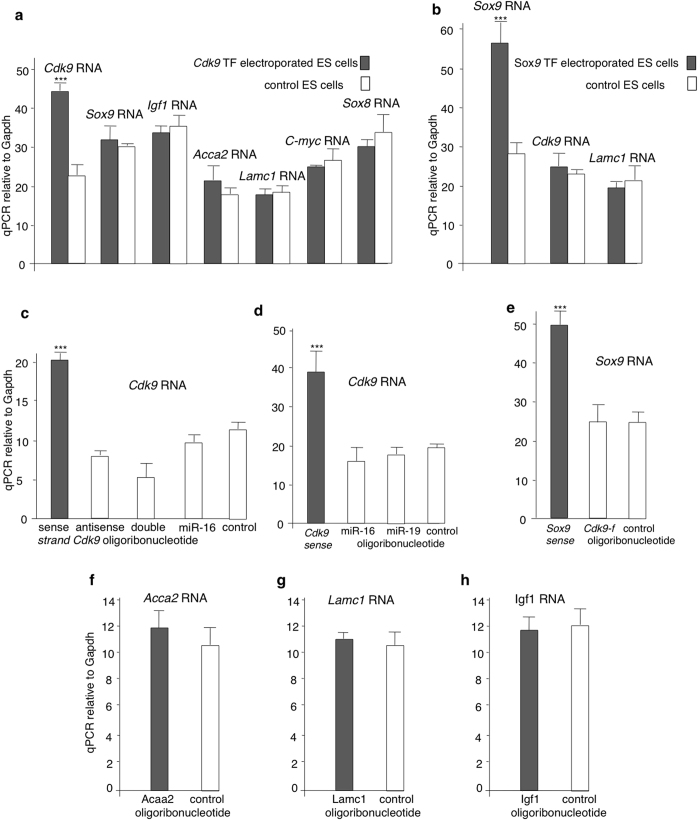
Not all genes are susceptible to RNA mediated gene activation. (**a**,**b**) Analysis of several genes by quantitative real time PCR assay revealed that the increased expression levels of mRNA following TF electroporation were specific to *Cdk9* and Sox9 (*n = 4*). (**c**) Levels of *Cdk9* expression following electroporation of *Cdk9* derived single (sense and antisense) and double-stranded transcript fragments in mass cultures of the ES cells (*n = 4*). (**d**,**e**) Among the several electroporated oligoribonucleotides only *Cdk9* and Sox9 derived transcript fragments could induce the corresponding RNAs, respectively. (**f**–**h**) Transcription induction was not observed in Acaa2, Lamc1, and Igf1 loci upon electroporation of the transcript fragment corresponding to the respective genes. Quantitative RT-PCR determination was performed after 2 days. Control: mock-electroporated cultures (*n = 4*). Data are mean ± S.E.M. ***p < 0.001.

To evaluate whether the observed RNA-mediated gene activation is specific, we electroporated ES cells with the *Cdk9*-f oligoribonucleotide and measured expression of several genes including *Cdk9, Sox9, Igf1, Acca2, Lamc1, C-myc,* and *Sox8* by quantitative real time PCR. Only *Cdk9* expression increased upon electroporation of the *Cdk9*-f oligoribonucleotide (Fig. 5a). The same was evident for *Sox9. Sox9* expression increased exclusively upon electroporation of the respective oligoribonucleotide (Fig. 5b). In further electroporation experiments, we confirmed that the transcriptional activation of *Cdk9* and *Sox9* is exclusively induced by the respective sense-strand oligoribonucleotides. Neither antisense nor double-stranded oligoribonucleotides or unrelated micro RNAs induced *Cdk9* or *Sox9* expression (Fig. 5c–e). To investigate whether RNA-mediated gene activation is a unique property of the *Cdk9* and *Sox9* loci, quantitative real time PCR assays were performed following electroporation of 22 bp sense transcript fragments of three other genes *Acca2, Lamc1*, and Igf1. No significant increase in the corresponding RNAs was evidenced (Fig. 5f–h). No antisense transcript was detected for these three genes (data not shown). Our results indicate that under our experimental conditions, not all genes are susceptible to transcriptional activation by non-coding RNAs.

### RNA-mediated programming of ES cell differentiation

**Figure 6:**
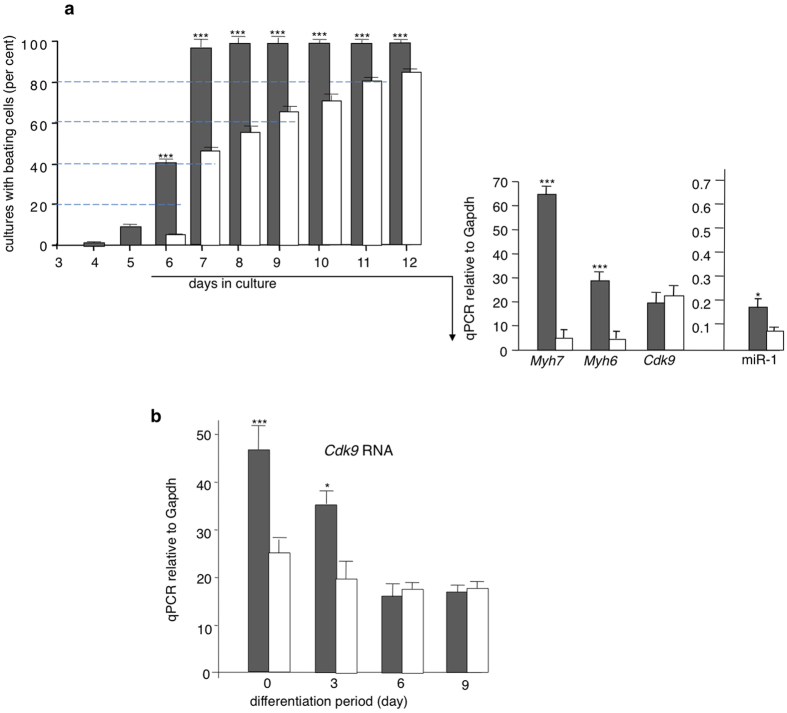
Efficient directed cardiac differentiation of ES cells following *Cdk9* induction by the small transcript fragment (TF). (**a**) Upper panel. Six sets of 48-well tissue culture plates were inoculated with cells from the embryoid bodies of hanging drop cultures (one hanging drop culture per well) in order to follow *in vitro* differentiation (3 plates for TF ES cells and 3 for control ES cells). The appearance of beating cells was monitored every day. Lower panel. Three sets of plates were independently seeded with cells from TF hanging drops and three for control ES cells, 20 hanging drops being transferred into each plate. At day 6 of differentiation, total RNA was extracted and qRT-PCR analysis of cardiac differentiation markers Myh6, Myh7, and miR-1 were performed. (**b**) *Cdk9* expression in differentiating cells returns to control values. Total RNA was extracted on days 0, 3, 6, and 9 during the cardiac differentiation period and *Cdk9* expression was examined by qRT-PCR. Dark shaded bars represent TF ES cells and white bars control ES cells. *p < 0.1, ***p < 0.001.

The morphology and growth characteristics of the RNA-modified ES cells were still those of the original ES line. They did not exhibit any morphological signs of differentiation, neither towards cardiac muscle nor other tissue-specific cell types. To confirm this observation, we investigated expression of two characteristic embryonic stem cell markers, *Nanog* and *Oct3*/4. Furthermore, we evaluated that transcripts characteristic for cardiac differentiation were not detectable (Table S1). However, these cells differentiated faster and more efficiently into cardiac muscle cells than the original ES line (“RNA-mediated programming”). According to well-established procedures^4^, embryoid bodies were generated from sense *Cdk9*-f-electroporated and control ES cells by culture in hanging drops. Cells were plated back on gelatin-coated plates 3 days later. Cardiac differentiation monitored by the appearance of beating cells progressed at a faster rate in the progeny of *Cdk9*-induced cells than in the original ES cells (Fig. 6a). Accordingly, quantitative RT-PCR determination of cardiac marker genes showed higher values than in control cultures. The level of miR-1, undetectable in freshly electroporated cells as well as in the original ES cells, was increased during differentiation as reported previously^12^. Interestingly, expression of *Cdk9* in differentiating subcultured cells had returned to control values by day 6 (Fig. 6b), but remained elevated after electroporation for 15 days in cells without subculturing (data not shown).

Injection of *miR-1* or *Cdk9-f*-electroporated ES cells into blastocysts resulted in increased expression of *Cdk9* in embryonic hearts at E18.5 (Suppl. Fig. 5a). Furthermore, injection of *Cdk9-f*-electroporated ES cells into blastocysts increased heart-to-body weight ratios (Suppl. Fig. 5b) and higher ventricular dimensions (Suppl. Fig. 5c) in embryos at E18.5. Electroporation of a pIRESneo-EGFP DNA/miR-1 construct in ES cells and subsequent transfer into blastocysts confirmed a specific higher GFP expression in embryonic hearts (Suppl. Fig. 6a), but not in liver, kidney, and lungs (Suppl. Fig. 6a,b). In these embryos, heart weights were higher, while kidney and lung weights were comparable to controls (Suppl. Fig. 6c). Histological analysis confirmed the increase in heart size (Suppl. Fig. 6d), which was due to cardiac hyperplasia as indicated by the increased nuclei count (Suppl. Fig. 6e) indicating that RNA programmed ES cells also contribute to the heart *in vivo*.

## Discussion

While our previous results indicated the possibility of RNA-mediated induction of gene expression in embryos, they did not allow full investigation regarding the mechanisms leading to augmentation of transcriptional activity. The scarcity of one-cell embryos made the search for molecular genetic mechanisms technically demanding or impossible because of lethality or sterility of animals. To overcome this problem, we developed cell culture systems that could be more amenable to molecular analyses. Because of a relatively high level of expression in ES cells and the availability of well-established protocols for cardiac differentiation *in vitro*, induction of *Cdk9* by a transcript fragment provides a convenient experimental system. The single-stranded sense transcript fragment homologous to the carboxy-terminal *Cdk9* exon induced transcription of the gene in ES cells significantly. This effect was not unique to ES cells, since a comparable increase in expression was evidenced upon electroporation of the same transcript fragment in embryonic fibroblasts and a human keratinocyte cell line.

Furthermore, we examined transcriptional levels following electroporation of ES cells with several transcript fragments corresponding to different regions of *Cdk9.* Interestingly, *Cdk9* induction was not observed upon electroporation of the oligoribonucleotides with a nucleotide sequence identical to that of the mRNA in the middle region of *Cdk9 (Cdk9*-b-e, Table 1) and of single stranded DNA oligonucleotides (Table 1). This convenient experimental system enabled us to outline the sequence of molecular events that result in augmentation of transcriptional activity. The requirement of *Argonaute* proteins for transcriptional activation indicates that Ago proteins charge the incoming small RNA fragments, and the RNAi machinery perceives the initial small RNA signal. Interestingly, natural antisense transcripts (NATs) complementary to the most 3′ and 5′ regions of the mRNA were detected and locus-specific induction of *Cdk9* expression identified following targeting of their antisense transcripts by short single stranded cognate transcript fragments. Indeed, hybridization of the electroporated oligoribonucleotide with antisense transcripts provides the signal for transcriptional activation. However, it is unlikely that simply the anti-sense transcript impedes progression of the transcription machinery as targeting of the 3′ antisense transcript resulted in increased RNA pol II binding to DNA in the promoter and 3′ UTR region. Thus, it seems likely that increased transcription is due to modification of the genomic locus.

Similar to a recent study where specific induction of the BDNF locus was investigated^13^, single stranded sense transcripts targeting natural antisense transcripts are sufficient to initiate an induction of *Cdk9* mRNA expression. In case of the mentioned study, additional significant changes in histone marks have been identified. Aside from the presence of endogenous antisense transcripts, the RNAi machinery requires *Argonaute* proteins. The observation that transcriptional activation was not detected in Ago-deficient cells indicates that RNAi was linked to transcriptional activation. Although numerous studies have reported that RNAi gene silencing acts by targeting sense transcripts, our study shows that RNAi gene activation operates by targeting antisense transcripts. This is in agreement with the report by Zhang *et al*.^14^ showing in Hela cells with a CMV-EGFP reporter gene system that antisense RNAs and Ago2 are involved in transcriptional activation^14^. We extend this finding to endogenous genes and provide evidence that not only long non-coding RNAs, but also small RNA fragments can induce gene activation.

Mechanistically, *Cdk9* antisense transcripts might form RNA-RNA duplexes with *Cdk9* mRNA as it has been described for BDNF^13^. Electroporation of sense oligoribonucleotides favors duplex formation between the sense oligoribonucleotide and the antisense transcript. This complex is processed by the RNAi machinery as indicated by the requirement of Ago proteins. Activation of the RNAi machinery may then result in changes in chromatin marks at the locus as described for several genes^10^,^13^,^14^, leading to increased transcription of the *Cdk9* locus (Fig. 2). Reduced expression of the antisense transcript in response to electroporation of the sense oligoribonucleotide (Fig. 3e) indicating destruction of the antisense transcript could represent a secondary phenomenon of the activation of the RNAi machinery. Although it is widely assumed that the RNAi machinery functions mainly in the cytoplasm, specific and potent activity has been demonstrated also in the nucleus^13^,^14^,^15^. Whether the RNAi machinery gains access to genomic DNA during cell division when the nuclear membrane is disrupted or whether a fraction of RNA-protein complexes is actively transported into the nucleus remains an open question.

As a functional consequence of the *Cdk9* locus activation, we observed an increased cardiac differentiation potential of the sense oligoribonucleotide electroporated ES cells. The modified ES cells overexpressing *Cdk9* maintained a pluripotent state when propagated in LIF containing medium. They expressed *Nanog* and *Oct4* and did not express cardiac-specific genes (Table 1). When cardiac differentiation was started, however, they exhibited a greater differentiation potential than the original ES cells. During this process, *Cdk9* expression returned to normal values, which might be related to the limited stability of the sense RNA fragments.

Interestingly, levels of miR-1 have been shown to increase during normal heart development and upon spontaneous myocardial differentiation of ES cells in 2-D culture^12^. Also in our experimental system of increased cardiac differentiation, miR-1 expression was increased. We show that a short pulse of sense transcript fragment induced over-expression is sufficient to create a “memory” for cardiac differentiation in ES cells. Also *in vivo,* reprogrammed ES cells injected in wild-type blastocysts contributed to heart development, which suggests that cells transiently exposed to small RNAs might become a novel tool for ES cell re-programming for regenerative medicine.

Our data are in agreement with the emerging view that short RNA derived from longer messages might not just be short degradation products, but could have specific functions as well^16^,^17^,^18^. A short t-RNA like structure has been shown to arise specifically from the long non-coding MALAT1 RNA^16^. Later, an important number of small and large RNAs generated from post-transcriptional processing of mature mRNAs have been identified^17^. Most recently, Pircher *et al*. showed that an mRNA-derived small RNA is able to bind and regulate ribosomes^18^. Thus, our identification of the requirement of endogenous antisense transcripts and Ago proteins to induce transcriptional activation by small sense RNAs contributes to the complexity of the picture.

## Conclusion

*Cdk9* transcript-derived oligoribonucleotides are capable to induce *Cdk9* expression in different cell systems. Requirements for Argonaute proteins and for endogenous antisense transcripts at the locus indicate that the inducer oligoribonucleotides are processed by the RNAi machinery. Induction of *Cdk9* resulted in efficient cardiac differentiation of ES cells *in vitro*. Injection of *Cdk9-f*-electroporated ES cells into blastocysts induced cardiac growth indicating that RNA-programmed ES cells contribute specifically to the heart *in vivo*.

## Methods

### Cell culture and RNA electroporation

Mouse AB1 ES cells were grown on mouse embryonic fibroblast (MEFs) feeders in standard ES culture medium. Briefly, ESCs were cultured on a feeder layer of Mitomycin C treated MEFs on 0.2% gelatin-coated cell culture dishes. Culture medium consisted of Dulbecco’s modified Eagle’s medium, 15% ES-grade fetal calf serum (FCS, Gibco), 1 mM sodium pyruvate (Gibco), 0.1 mM non-essential amino acids (Gibco), 0.1 mM β-mercaptoethanol (Sigma), 100 U/ml penicillin and 0.1 mg/ml streptomycin and 1000 U/ml leukemia inhibitory factor (LIF). Cardiac differentiation of ESCs was induced according to established procedures^4^. Mouse embryonic fibroblasts (MEFs) and human keratinocyte cell lines were cultured in DMEM containing 10% FCS. *Ago1*^−/−^, *3*^−/−^, *4*^−/−^ ES cells were kindly provided by X. Wang (Department of Biochemistry, Northwestern University, Evanston, USA). Electroporation was performed 1–2 days after plating (5 × 10^6^ cells per plate) with 5 μg RNA using the Bio-Rad Gene Pulser apparatus (400 V, 250 F).

### Quantitative RT-PCR RNA analysis

RNA was extracted using the Trizol Reagent (Invitrogen). 0.5 μg RNA samples were reverse transcribed to cDNA using random hexamer primers and MLV reverse transcriptase (Invitrogen). q-PCR was performed using the ‘Platinum^®^ SYBR^®^ Green qPCR SuperMix-UDG’ kit (Invitrogen). Oligoribonucleotides and their FITC- and Cy3-labelled derivatives were obtained from SIGMA-PROLABO. The average threshold (Ct) was determined for each gene and normalized to Gapdh mRNA level as internal normalization control. Sequences of deoxyribo-nucleotide primers are provided in Table S2.

Strand-specific RT-PCR was performed to detect potential locus-specific antisense transcripts. Specific 18–20 single stranded oligonucleotides complimentary to potential antisense transcripts were designed to target only antisense RNA strands. Thus, in the reverse transcription step, complementary DNA (cDNA) was generated from antisense templates. The reverse transcriptase was omitted for negative controls.

### Run-on assay of transcriptional activity

Assays were performed according to Patrone *et al*.^19^ by q-PCR monitoring the incorporation of biotin-labeled triphosphate (biotin-16-UTP, 11388908910, Roche Applied Science) into RNA isolated on Streptavidin Magnetic Particles (11641778001, Roche Applied Science).

### Western blot analysis

Total lysates from cell cultures were prepared, electrophoresed, and blotted as described^20^. The following antibodies were used for immunodetection: polyclonal anti-CDK9 antibody from rabbit (H-169, sc-8338, Santa Cruz Biotechnology) in a 1:500 dilution in PBS, 2.5% Blotto, 0.05% Tween-20, mouse monoclonal anti-gapdh (T6199, Sigma) 1:2000, peroxidase-coupled goat anti-rabbit secondary antibody (Santa Cruz Biotechnology) 1:10,000 and peroxidase-coupled rabbit anti-mouse secondary antibody (Santa Cruz Biotechnology) 1:10,000.

### Northern blot analysis

Northern blot analysis was performed according to standard methods. Briefly, 6 μg total RNA extracted from cell cultures was loaded onto a 12% denaturing polyacrylamide gel and electrophoresed until the bromophenol blue marker reached the bottom of the gel. The separated RNA was electrotransferred to a Hybond N+ membrane (Amersham). Hybridization was carried out in the presence of ^32^P-end-labeled DNA probes. 440 and 594 nucleotide probes for *Cdk9* and *Gapdh*, respectively, were generated by PCR using the following oligonucleotides: *Cdk9* forward, 5′-TGAAGCTGGCAGATTTTGGG-3′; *Cdk9* reverse, 5′-GCATCGTCACTGTCAATCCG-3′); Gapdh forward, 5′-TTCCTATAAATACGGACTGCAG-3′; Gapdh reverse, 5′-GTTCCTAATACTTAAGACTCCG-3′.

### Chromatin immunoprecipitation (CHIP) assay

Chromatin immunoprecipitation (CHIP) assay was carried out according to the protocol of the ChIP Assay Kit (Millipore cat. 17–295). Briefly, at least 1 × 10^6^ cells were growth in 100 mm dishes, and were cross-linked by adding formaldehyde to a final concentration of 1% and incubated at room temperature for 10 minutes. Cells were washed twice, using ice cold PBS containing protease inhibitors (1 mM phenylmethylsulfonyl fluoride (PMSF), 1 μg/ml aprotinin, and 1 μg/ml pepstatin. The scraped cell suspension was transferred to a conical tube and centrifuged 4 min at 2000 rpm at 4 °C. Cells were washed with PBS and re-suspended in ChIP lysis buffer (1% SDS, 10 mM EDTA, 50 mM Tris-HCl pH 8.0) with added protease inhibitors (inhibitors: 1 mM PMSF, 1 μg/ml aprotinin, and 1 μg/ml pepstatin A). After 10 minutes incubation on ice, cells were sonicated to shear DNA to lengths between 200 and 1000 base pairs. Preparation of chromatin fragments, immunoprecipitation, and DNA recovery were performed as described in the manufacturer instructions. The antibody directed against RNA polymerase II was obtained from Santa Cruz Biotechnology (N-20, sc-899, Santa Cruz Biotechnology). The following primers were used: *Cdk9* P2 promoters, *Cdk9*-P2F: 5′-ATGCAGCGGGACGCACCG-3′ and *Cdk9*-P2R: 5′-GGGAGCCGGAGCTGCAGAGG-3′; *Cdk9* P1 promoters, *Cdk9*-P1F: 5′-GGGAACTACAAGTCCCAGG-3′ and Cdk9-P1R: 5′-CACTCCAGGCCCCTCCGCGG-3′; *Cdk9* 3′ UTR primers, UTR-F: 5′-TTGAGATTTTCTCCTCCAGTAC-3′ and UTR-R: 5′-TTGAGATTTTCTCCTCCAGTAC-3.

### ES cells injection of blastocysts

According to standard methods, ES cells were injected in 3.5 days blastocysts after electroporation of *Cdk9* sense transcript fragment (Cdk9-f: 5′-GAUUUUCUCCUCCAGUACAUAU-3′), or microRNA-1 (miR-1: 5′-UGGAAUGUAAAGAAGUAUGUAU-3′), or a pIRESneo-EGFP DNA/miR-1 construct. As controls, blastocysts were microinjected with mock-electroporated ESCs. For studies of embryos during gestation, the embryos were re-implanted in the uterine horns of the foster mother. Investigations were conducted in accordance with French and European rules for the care and use of laboratory animals and approved by the local ethical committee (Ciepal). Embryos were dissected at embryonic day 18.5, organ weighted and hearts prepared for histological analysis as described^2^.

### Statistical analysis

Data are expressed as means ± S.E.M. Differences between two groups were tested using the Mann-Whitney test for nonparametric samples. A *p* value less than 0.05 was considered statistically significant.

## Additional Information

**How to cite this article:** Ghanbarian, H. *et al*. Small RNA-directed epigenetic programming of embryonic stem cell cardiac differentiation. *Sci. Rep.* **7**, 41799; doi: 10.1038/srep41799 (2017).

**Publisher's note:** Springer Nature remains neutral with regard to jurisdictional claims in published maps and institutional affiliations.

## Supplementary Material

Supplementary Data 1

## Figures and Tables

**Table 1:** Oligoribonucleotides in ES electroporation experiments.

Designation	Gene and accession number	Position in the gene (nt)	Sequence (5′-3′)
miR-1	mmu-miR-1 (MIMAT0000123)		UGGAAUGUAAAGAAGUAUGUAU
miR-16	mmu-miR-16 (MIMAT0000527)		UAGCAGCACGUAAAUAUUGGCG
miR-19	mmu-miR-19 (MIMAT0000651)		UGUGCAAAUCUAUGCAAAACUGA
Cdk9-a	Cdk9 (NT_039206)	Intron 1 (10165433–10165457)	GAGUAAGCAGAGGCCGGGAGGAGG
Intron 1(DNA)		Intron 1 (10165433–10165457)	GAGTAAGCAGAGGCCGGGAGGAGG
Cdk9-b		Intron 2 (10167062–10167084)	CUCUGUGUAGCCCUGGCUGACC
Cdk9-c		Exon 3 (10167231–10167253)	GAACCUAAUUGAGAUUUGUCGG
Exon 3(DNA)		Exon 3 (10167231–10167253)	GAACCTAATTGAGATTTGTCGG
Cdk9-d		Exon 4 (10167604–10167626)	UAGUCAAGUUCACGUUGUCUGA
Exon 4(DNA)		Exon 4 (10167604–10167626)	TAGTCAAGTTCACGTTGTCTGA
Cdk9-e		Exon 5 (10167912–10167934)	GUGUGGUGACAUUGUGGUACCG
Exon 5(DNA)		Exon 5 (10167912–10167934)	GTGTGGTGACATTGTGGTACCG
Cdk9-f		Exon 7 (10169886–10169908)	GAUUUUCUCCUCCAGUACAUAU
Exon 7(DNA)		Exon 7 (10169886–10169908)	GATTTTCTCCTCCAGTACATAT
siRNA		Intron 6 (10168731–10168750)	AUAGUAGAGAGUCCACUGGTT
Sox9	(NT_165773.2)	Exon 3 (11454144–11454166)	GUUCCUAGAACAUUCACUGUGC
Acaa2	(NT_039674)	Exon 2 (71779229–71779249)	GCGGAAUAGCUGAGCUUCGC
LamC1	(NT_078297)	Exon 20 (67787375–67787386)	GAGAUCGCCUCCAGGGAGCTC
Sox8	(NT_039649)	Exon 2 (11915752–11915774)	GGCAACCUUGGAUUCUAGAGUG
Igf1	(NT_039500)	Exon 6 (29658914–29658936)	UGCUUGCUCACCUUCACCAGCU
