# Supplementary material for: Small RNA-directed epigenetic programming of embryonic stem cell cardiac differentiation
Source: Sci Rep. 2017 Feb 6;7:41799. doi: 10.1038/srep41799 (PMC5292948; doi:10.1038/srep41799)
Supplement: Supplementary Data 1 [file srep41799-s1.doc]

**Small RNA-directed epigenetic programming of embryonic stem cell cardiac differentiation**

Hossein Ghanbarian^1^, Nicole Wagner^2,3^, Jean-François Michiels^3,4^, François Cuzin^3^, Kay-Dietrich Wagner^2,3^*, and Minoo Rassoulzadegan^3^*

Supplementary Information

**Supplementary Figure 1: Genomic localization of the *Cdk9* sense and antisense transcripts on chromosome 2.** Solid boxes show exons while arrows depict introns and the direction of transcription. *Cdk9* antisense RNAs are transcribed from the opposite DNA strand as that of the coding *Cdk9* mRNA. The 3’ antisense transcript is covering the 3’ un-translated region, exon 7, and a section of the last intron of *Cdk9*. The 5’ antisense transcript complements the 5’ regions of the coding sequence including the P1 promoter and exon 1.

**Supplementary Figure 2: Antisense transcription at the human Cdk9 locus.** Regions analyzed for the presence of antisense transcripts throughout the human Cdk9 gene. Top: schematic representation of the locus with exons shown as closed bars. The positions of the regions analyzed are indicated. Bottom: detection of antisense RNA transcripts by strand-specific RT-PCR reactions using primers listed in Table S2. All assays were performed in duplicate, „RT-„: reverse transcriptase omitted.

**Supplementary Figure 3: Antisense transcription at the *Sox9* locus.** Regions analyzed for the presence of antisense transcripts covering the *Sox9* gene. A schematic representation of the locus is shown on top. Eight, nine, and ten indicate positions of the regions analyzed. Primer sequences are provided in Suppl. Table 1. The lower panel depicts detection of antisense RNA transcripts by strand-specific RT-PCR. All assays were performed in duplicate.

**Supplementary Figure 4: Silencing of the antisense transcript induces *Cdk9* expression.** The siRNA was designed against the intron 6 part of the antisense transcript. Detection of antisense RNA transcripts was performed by strand-specific quantitative RT-PCR. Quantitative RT-PCR determination of *Cdk9* RNA was performed 2 days after electroporation of the siRNA. Electroporation of the Cdk9-f oligoribonucleotide served as positive control (*n*=4 each). Data are mean±S.E.M. *p<0.05, **p<0.01, ***p <0.001.

**Supplementary Figure 5: Reprogrammed ES cells induce cardiac growth *in vivo.* (a)** Increased expression of *Cdk9* was detected by quantitative RT-PCR in hearts at embryonic day 18.5 (E18.5). ES cells were injected in 3.5 days blastocysts after electroporation of a *Cdk9* sense transcript fragment (Cdk9-f) or microRNA-1. As controls, blastocysts were microinjected with mock-electroporated ESCs (*n=4* each). **(b)** Heart to body weight ratios from embryos at E18.5. ES cells were injected in 3.5 days blastocysts after electroporation of a *Cdk9* sense transcript fragment (Cdk9-f). As controls, blastocysts were microinjected with mock-electroporated ESCs (*n=4* each). **(c)** Histological comparison of embryonic hearts after blastocyste injection of re-programmed (left panel) and control ES cells (right panel) at day 18.5 of embryonic development (*n=4* each). Haematoxylin-eosin staining of paraffin sections indicate increased heart size in embryos injected with the re-programmed ES cells. Scale bars indicate 500μm. ***p <0.001.

**Supplementary Figure 6: miR-1 reprogrammed ES cells contribute specifically to the heart *in vivo.* (a)** GFP expression in embryos at E18.5. ES cells were injected in 3.5 days blastocysts after electroporation of a pIRESneo-EGFP DNA/miR-1 construct. As controls, blastocysts were microinjected with mock-electroporated ESCs (*n*=*4* each). GFP was detected by fluorescence microscopy on cryostat sections. **(b)** Quantitative measurement of GFP expression in organs from embryos at E18.5 by quantitative RT-PCR. ES cells were injected in 3.5 days blastocysts after electroporation of a pIRESneo-EGFP DNA/miR-1 construct. As controls, blastocysts were microinjected with mock-electroporated ESCs (*n*=*4* each). **(c)** Heart, lung, and kidney to body weight ratios from embryos at E18.5 following the same protocol as in **(b). (d)** Histological comparison of embryos after blastocyste injection of re-programmed (left panel) and control ES cells (right panel) at day 18.5 of embryonic development (*n=4* each). Haematoxylin-eosin staining of paraffin sections indicate increased heart size and higher nuclei number in embryos injected with the re-programmed ES cells. Scale bars indicate 500μm (upper panel) and 50μm, respectively. **(e)** The nuclei count was determined for both groups of embryos on haematoxylin-eosin stained paraffin sections on the left ventricular free wall *(n=4* each). *p<0.05, **p<0.01, ***p <0.001.

Supplementary Table 1. TF electroporated ES cells express ES markers, but not cardiac-specific genes

| RNA^$^ | Control ES cells | |  | TF ES cells | |  |
| --- | --- | --- | --- | --- | --- | --- |
|  | Standard  culture condition | Cardiac  differentiation  day 6 |  | Standard  culture condition | Cardiac  differentiation  day 6 |  |
| ES cell markers | | | | | |  |
| Oct4 | 5.8 ± 1.2 | < 0.05 |  | 7.2 ± 1.9 | < 0.05 |  |
| Nanog | 10.2 ± 1.6 | 0.8 ± 0.3 |  | 12.1 ± 1.2 | 0.3 ± 0.1 |  |
| Cardiomyocyte markers | | | | | |  |
| miR–1 | < 0.05 | 0.1 ± 0.02 |  | < 0.05 | 0.2 ± 0.04 |  |
| Myh7 | < 0.05 | 5.8 ± 2 |  | < 0.05 | 64.8 ± 5 |  |
| Myh6 | < 0.05 | 3.5 ± 3 |  | < 0.05 | 30 ± 4 |  |
| ^$^ q-PCR values relative to *Gapdh* | | | | | |  |

**Supplementary Table 2**. Primers for RT -PCR analysis

| Gene | Designation | Position in the gene | Sequence 5’-3’ |
| --- | --- | --- | --- |
| mCdk9 | 0 | 5’UTR-Exon 1 | F: CCGCGGAGGGGCCTGGAGTG |
| (NT_039206) |  |  | R: AACGGGCATTCCACCGAGTCGTAC |
|  | 1 | Exon 1-Intron 1 | F: GTACGACTCGGTGGAATGCCCGTT |
|  |  |  | R: CCGAATGTGCCTTGGCCGAT |
|  | 2 | Exon 2-Intron 2 | F: TAAAGCCAAGCACCGTCAGA |
|  |  |  | R: GCCAAATTCCTTGTTCCATGG |
|  | 3 | Exon 3-Intron 3 | F: TCTACAGCTCCTAAAACATGAG |
|  |  |  | R: GCAGGTGGACCTCTAAGT |
|  | 4 | Exon 4-Intron 4 | F: GGTGTTTGACTTCTGTGAGCA |
|  |  |  | R: AGCTCAGTCAGGTTTCATAGC |
|  | 5 | Exon 5-Intron 5 | F : GGGTCCTGAAGCTGGCAGAT |
|  |  |  | R: CCGGGAGTGCCTCTTAAGC |
|  | 6 | Exon 6-Intron 6 | F: TGCATCATGGCAGAGATGTG |
|  |  |  | R: CAAACAAACAAACAAACAAACTG |
|  | 7 | Exon 7-3’UTR | F: AACCAGACGGAATTTGAACG |
|  |  |  | R: AGGAGAAAATCTCAAATGTCC |
| hCDK9 | 0 | 5’UTR-Exon 1 | F: CCTCTCGGGAACTACAAGTC |
|  |  |  | R: TCGTATTTGGAAACTTCATCAC |
|  | 1 | Exon 1-Intron 1 | F: GTTTCCAAATACGAGAAGCTC |
|  |  |  | R: CAGTCCCTTCTCCTCACTTG |
|  | 2 | Intron 1- Exon 2 | F: CAAGTGAGGAGAAGGGACTG |
|  |  |  | R: CATCAGCACCTTCTTCAGAG |
|  | 3 | Exon 2-Intron 2 | F: TGAAGAAGGTGCTGATGGA |
|  |  |  | R: ACGATGCATGCATCAACTC |
|  | 4 | Exon 3-Intron 3 | F: GGAGATCAAGATCCTTCAGC |
|  |  |  | R: TTTCTGAGTGCCTCTTCTGG |
|  | 5 | Exon 4- Exon 5 | F: ATGTTTTGGTCAAGTTCACG |
|  |  |  | R: CGAGTGATAAGCACATTAGCA |
|  | 6 | Intron 6 - Exon 7 | F: TTTATGAAGGGATAAGCCAC |
|  |  |  | R: AGAAGAAGTCGTGGTTGAGG |
|  | 7 | Exon 7-3’UTR | F: TTCTTCTGCTATGTGACTTGC |
|  |  |  | R: ACCTCCAGTGACTTGACAATC |
|  | Cdk9-F | Exon2-Exon3 | F: TAAAGCCAAGCACCGTCAG |
|  | Cdk9-R |  | R: GATTTCCCTCAAGGCTGTGAT |
|  | Cdk9-AS-F | 3’UTR | F : TTGAGATTTTCTCCTCCAGTAC |
|  | Cdk9-AS-R |  | R : AAGTCACAGTGGGTGAGCCT |
|  | P2-F | P2 Promoter | F: ATGCAGCGGGACGCACCG |
|  | P2-R |  | R: GGGAGCCGGAGCTGCAGAGG |
|  | P1-F | P1 Promoter | F: GGGAACTACAAGTCCCAGG |
|  | P1-R |  | R: CACTCCAGGCCCCTCCGCGG |
| Sox9 | 8 | Exon 1-Intron 1 | F: CTGAAGGGCTACGACTGGAC |
|  |  |  | R: CTGCATCCTGGACCAAAGAC |
|  | 9 | Exon 2-Intron 2 | F: GAAAGACCACCCCGATTACA |
|  |  |  | R: GGGGGATGCAGAGAATAGTG |
|  | 10 | Intron 2-Exon 3 | F: ACCAATACTTGCCACCCAAC |
|  |  |  | R: CTCAGCTGCTCCGTCTTGAT |
|  | Sox9-F | Exon2-Exon3 | F: GATCTACAGGCCTCTACCAG |
|  | Sox9-R |  | R: TGCAGCCTCCTGGGTATGAG |
| GFP |  |  | F: GACCCTGAAGTTCATCTGCACCA |
|  |  |  | R: CTTGTAGTTGCCGTCGTCCTTGA |
| Gapdh |  |  | F:TGTCCGTCGTGGATCTGAC |
| (NM_008084) |  |  | R: CCTGCTTCACCACCTTCTTG |
| Nkx2.5 |  |  | F: GACGTAGCCTGGTGTCTCG |
| (NM_008700) |  |  | R: GTGTGGAATCCGTCGAAAGT |
| myh6 |  |  | F: CCAAGACTGTCCGGAATGA |
| (NC_000080.5) |  |  | R: TCCAAAGTGGATCCTGATGA |
| myh7 |  |  | F: GGCCTCCATTGATGACTCTG |
| (NC_000014.8) |  |  | R: CGCCTGTCAGCTTGTAAATG |
| Igf1 |  |  | F : AAATCAGCAGCCTTCCAACT |
| (NM_010512.4) |  |  | R : GTGAAGGTGAGCAAGCAGAG |
| Acca2 |  |  | F : GTGTGGAGGAACAGAGAGCA |
| NC_000962.3 |  |  | R : TGCCCACAAAGTATCTTCCA |
| Lamc1 |  |  | F : ATGCCAGCGAGTGTGTAAAG |
| NM_010683.2 |  |  | R : ATTGAAGAAAGGCAGGCACT |
| Sox8 |  |  | F: GAAGCTGGCAGACCAGTACC |
| NC_000083.6 |  |  | R: GGTCTCTTCTCGCTCTCGTTC |
